# Rejuvenating Effector/Exhausted CAR T Cells to Stem Cell Memory–Like CAR T Cells By Resting Them in the Presence of CXCL12 and the NOTCH Ligand

**DOI:** 10.1158/2767-9764.CRC-21-0034

**Published:** 2021-10-19

**Authors:** Makoto Ando, Taisuke Kondo, Wataru Tomisato, Minako Ito, Shigeyuki Shichino, Tanakorn Srirat, Setsuko Mise-Omata, Kensuke Nakagawara, Akihiko Yoshimura

**Affiliations:** 1 Department of Microbiology and Immunology, Keio University School of Medicine, Shinjuku-ku, Tokyo, Japan.; 2 Oncology Research Laboratories I, Daiichi Sankyo Co., Ltd., Shinagawa-ku, Tokyo, Japan.; 3 Medical Institute of Bioregulation, Kyushu University, Higashi-ku, Fukuoka, Japan.; 4 Division of Molecular Regulation of Inflammatory and Immune Diseases, Research Institute for Biomedical Sciences, Tokyo University of Science, Noda City, Chiba, Japan.

## Abstract

T cells with a stem cell memory (T_SCM_) phenotype provide long-term and potent antitumor effects for T-cell transfer therapies. Although various methods for the induction of T_SCM_-like cells *in vitro* have been reported, few methods generate T_SCM_-like cells from effector/exhausted T cells. We have reported that coculture with the Notch ligand–expressing OP9 stromal cells induces T_SCM_-like (iT_SCM_) cells. Here, we established a feeder-free culture system to improve iT_SCM_ cell generation from expanded chimeric antigen receptor (CAR)-expressing T cells; culturing CAR T cells in the presence of IL7, CXCL12, IGF-I, and the Notch ligand, hDLL1. Feeder-free CAR-iT_SCM_ cells showed the expression of cell surface markers and genes similar to that of OP9-hDLL1 feeder cell–induced CAR-iT_SCM_ cells, including the elevated expression of SCM-associated genes, *TCF7*, *LEF1*, and *BCL6*, and reduced expression of exhaustion-associated genes like *LAG3*, *TOX*, and *NR4A1*. Feeder-free CAR-iT_SCM_ cells showed higher proliferative capacity depending on oxidative phosphorylation and exhibited higher IL2 production and stronger antitumor activity *in vivo* than feeder cell–induced CAR-iT_SCM_ cells. Our feeder-free culture system represents a way to rejuvenate effector/exhausted CAR T cells to SCM-like CAR T cells.

**Significance:**

Resting CAR T cells with our defined factors reprograms exhausted state to SCM-like state and enables development of improved CAR T-cell therapy.

## Introduction

Adoptive T-cell transfer therapy is an emerging field that involves the isolation and reinfusion of T lymphocytes into patients to treat cancer and other diseases, including autoimmune diseases (1). Chimeric antigen receptor T (CAR T) cells provide dramatic therapeutic effects for hematologic cancers and have led to complete remission in patients whose cancer has failed to respond to standard therapy (2, 3). However, depending on both the specific type of hematologic cancer and the patient, low responses and high rates of relapse have been observed (3–6). This resistance to CAR T-cell therapy has been shown to be correlated with decreased early memory cell fraction in infusion products and increased T-cell exhaustion (7). An early memory phenotype contributes to long-lasting and potent antitumor immunity in not only CAR T cells but also tumor-infiltrated T cells (8, 9).

T cells with a stem cell memory phenotype (T_SCM_) are a subset of memory T cells. T_SCM_ cells express both naïve T-cell markers, such as CD45RA, CCR7, CD62L, CD127, and central memory T cell (T_CM_) markers, such as CD27, CD28, CD95 and CD122 (10). T_SCM_ cells possess high self-renewal ability, multipotency, and long-term persistency; therefore, they have been expected to overcome the therapeutic limitations of adoptive immunotherapies, such as poor persistence and the lack of a long-lasting immune response. The potent antitumor activity of T_SCM_ cells also appears to be dependent on fatty acid oxidation (FAO)-dependent mitochondrial oxidative phosphorylation (OXPHOS; ref. 11). Fraietta and colleagues reported that early memory-related genes with reduced glycolysis signatures were enriched in CAR T cells from complete responding patients with chronic lymphocytic leukemia compared with nonresponding patients (12). Terminally differentiated effector T cells are more dependent on glycolysis, which results in the accumulation of oxidative stress and DNA damage (13, 14). Therefore, OXPHOS-dominant metabolism is an important feature as T_SCM_ cells ensure their longer persistence and a high antitumor ability (14, 15).

Currently, various methods have been reported to generate less differentiated T cells from naïve T cells by stimulating them with T-cell receptor (TCR) signals in the presence of a GSK3β inhibitor; TWS119 (16, 17), an Akt inhibitor (18–20), rapamycin (21), and metabolic state–modifying reagents (22, 23). On the other hand, we have reported that T_SCM_-like cells can be induced by coculturing activated/exhausted CD8^+^ T cells with OP9 feeder cells (FC) that express the Notch ligand, human Delta-like 1 (OP9-hDLL1), which we named induced T_SCM_ (iT_SCM_; refs. 24, 25). We also reported that this method converts human-activated CD8^+^ CAR T cells to SCM-like CAR T cells (CAR-iT_SCM_), which showed superior proliferative capacity, better resistance to cell death, *in vivo* persistence, and more potent antitumor effects compared with conventional CAR T cells (11). However, long-term coculture with mouse FCs may not be suitable for clinical applications due to the possibility of contamination with xenogeneic components and technical complications. In this study, we demonstrated that feeder-free CAR-iT_SCM_ (FF CAR-iT_SCM_) cells could be generated by resting activated CAR T cells under IL7 + IGF-I + CXCL12 + Notch ligand culture conditions. FF CAR-iT_SCM_ cells exhibited early memory phenotypes similar to FC-induced CAR-iT_SCM_ (FC CAR-iT_SCM_) cells. FF CAR-iT_SCM_ cells showed longer persistence and higher antitumor effects than conventional CAR T cells, even better than FC CAR-iT_SCM_ cells. We propose a novel strategy to rejuvenate effector/exhausted T cells to iT_SCM_ cells with high antitumor ability.

## Materials and Methods

### Mice

Male and female NOD.Cg-*Prkdc^scid^Il2rg^tm1Wjl^*/Szj (NSG) mice were purchased from Charles River Laboratory Japan. All mice were kept in specific pathogen–free facilities at Keio University (Tokyo, Japan). All experiments using mice were approved by the Institutional Animal Care and Use Committee (IACUC; approval number 08004) of Keio University (Tokyo, Japan) and were performed according to IACUC guidelines.

### Cell Lines

OP9 (RCB1124), TSt-4 (RCB2116), NIH 3T3 (RCB2767), NALM6 (RCB1933), and K562 (RCB0027) cells were purchased from RIKEN Bio Resource Center (Tsukuba, Ibaraki, Japan) within 10 years. OP9, TSt-4, and NIH 3T3 cells were cultured in minimum essential medium-alpha modification (αMEM; Thermo Fisher Scientific) supplemented with 20% FBS (Sigma-Aldrich) and 1% penicillin-streptomycin (Nacalai Tesque). NALM6 and K562 cells were cultured in RPMI1640 (Nacalai Tesque) supplemented with 10% FBS and 1% penicillin–streptomycin as described previously (13). OP9-hDLL1 and K562-hCD19 cells have been described previously (11, 26). HEK293T and Expi293F cells were purchased from (ATCC) and Thermo Fisher Scientific within 10 years, respectively. These cells were cultured in DMEM (Nacalai Tesque) supplemented with 10% FBS and 1% penicillin–streptomycin as described previously (1). All cell lines were regularly tested for *Mycoplasma* contamination using BioMycox Mycoplasma PCR Detection Kit (CellSafe) and were frozen down at early passages (<7) and used in the experiments within five passages after thawing. These cells were not further authenticated by our laboratory; however, routine confirmation of *in vitro* growth properties, morphology, and transfection efficiency (HEK293T and Expi293F) provided evidence of correct cell identity.

### Isolation, Transduction, and Expansion of Human CD8^+^ T Cells

Peripheral blood mononuclear cells (PBMC) were isolated via the density gradient centrifugation method from peripheral blood. Peripheral blood was obtained from eight healthy donors. All human studies were approved by the Institutional Review Board of the Keio University School of Medicine (Tokyo, Japan; approval number 20120039), and written informed consent was obtained from all participants.

Human CD8^+^ T cells were separated from PMBCs using a CD8^+^ T Cell Isolation Kit (Miltenyi Biotec). Isolated CD8^+^ T cells were activated with Dynabeads Human T-Activator CD3/CD28 (Thermo Fisher Scientific) and IL2 (30 ng/mL; PeproTech) in RPMI1640 medium supplemented with 10% AB serum (Innovative Research) for 6 days as described previously (11). Twenty-four hours after T-cell activation, the anti-CD19 CAR flanked with Venus transgene was transduced with a retrovirus into the CD8^+^ T cells via spin infection (2,500 rpm, 35°C, 2 hours), in the presence of polybrene (5 μg/mL) as described previously (1, 11). Eighteen hours after the spin infection, the cells were transferred into the T-cell media to remove both the retrovirus and polybrene. On day 6, CD8α^+^Venus^+^ cells were sorted by a FACSAria III cell sorter (BD Biosciences) and expanded by coculture with 40 Gy–irradiated K562-hCD19 (E:T ratio = 4:1) for 6 to 8 days.

We have used K562-hCD19 cells to achieve a high expansion of antigen-specific CAR T cells, which is suitable for basic analysis such as gene expression analysis and marker expressions. We confirmed similar iT_SCM_ induction after expansion of FACS-sorted CAR^+^ T cells with anti-CD3/CD28 beads, thus iT_SCM_ induciton can be applied to CAR T cells expanded by Good Manufacturing Practice (GMP)-compatible anti-CD3/CD28 beads.

### Plasmid Construction and Retroviral Transduction

A transgene encoding a CD19-specific CAR with 4–1BB/CD3z domains was synthesized and subcloned into the retroviral vector pMEI-5-MCS or pMEI-5-MCS-IRES2-Venus. The retrovirus was prepared as described previously (1).

For the hDLL1-hIgG1-Fc fusion protein expression vector, the signal peptide and extracellular domain of human DLL1 were subcloned into the pcDNA3-hIgG1-Fc vector. The pcDNA3-hIgG1-Fc vector was a kind gift from Dr. Kon (Fukuyama University, Hiroshima, Japan). To generate the hDLL1-Fc protein, the hDLL1-Fc expression vector was transfected into Expi293F cells. Sixteen hours after transfection, the transfected Expi293F cells were washed twice and cultured in serum-free DMEM/F-12 (Nacalai Tesque) supplemented with 0.5% BSA, 10 mmol/L HEPES, 2 mmol/L l-glutamine, 1 × NEAA, 1% penicillin–streptomycin, 1 mmol/L sodium pyruvate, 1 × Insulin-Transferrin-Selenium-Ethanolamine (Gibco), and Chemically Defined Lipid Concentrate (1:1,000 dilution; Gibco) for three days. The hDLL1-Fc–containing culture medium was collected, filtered through a 0.22-μm filter to remove cellular debris, and stored at 4°C.

### CAR-iT_SCM_ Induction by OP9-hDLL1 FCs

The CD8α^+^CD45RA^−^CD45RO^+^ cells were sorted as fully activated CAR T cells and were cocultured with OP9-hDLL1 cells in the presence of IL7 (10 ng/mL) in αMEM for 10 days as described previously (11, 26).

### CAR-iT_SCM_ Induction by the FF Culture System

To introduce Notch signaling into the T cells, protein A (4 μg/cm^2^; Sigma-Aldrich) and human fibronectin (FN; 2 μg/cm^2^; FUJIFILM Wako Pure Chemical Corporation) were coated onto a nontreated tissue culture plate (Falcon) at 4°C overnight. The protein A/FN–coated plates were blocked with Hank Balanced Salt Solution (HBSS) supplemented with 2% BSA, at room temperature for 45 minutes. The hDLL1-Fc–containing Expi293F culture supernatant was subsequently incubated on this plate at 37°C for two hours. The plate was washed twice and was used as the hDLL1-Fc–coated plate for the FF culture system for CAR-iT_SCM_ induction. The CD8α^+^CD45RA^−^CD45RO^+^ cells were sorted as fully activated CAR T cells were cultured in αMEM supplemented with IL7 (10 ng/mL; PeproTech), CXCL12 (100 ng/mL; PeproTech), and IGF-I (50 ng/mL; PeproTech) on the hDLL1-Fc–coated plate for 10 days. The other growth factors CCL2 (100 ng/mL), IL6 (10 ng/mL), TSLP (20 ng/mL), SCF (50 ng/mL), FLT3 L (20 ng/mL), Osteopontin (100 ng/mL), TPO (50 ng/mL), VEGF-A(20 ng/mL), TGFβ (2ng/mL), IFNβ (100 ng/mL), Gas6 (300 ng/mL), Leptin (300 ng/mL), R-spondin-3 (20 ng/mL), Tenascin C (1 μg/mL), and Pleiotrophin (20 ng/mL) were purchased from PeproTech.

As for FN, we have tried other extracellular matrix proteins such as ICAM-I and StemSpan Lymphoid Differentiation Coating Material (STEMCELL Technologies), but they were not as effective as fibronection for promoting CAR-iT_SCM_ formation. Retronectin (Takara) is as effective as FN, but we selected FN because FN is cost effective.

### Preparation of Various Conditioned Media from Stromal Cells

Once the OP9, OP9-hDLL1, TSt-4, and NIH 3T3 cells were approximately 80% confluent, we replaced the plate with fresh medium. Two days after the medium was changed, we collected the culture supernatant as the conditioned medium (CM). The CM was passed through a 0.45-μm filter to remove cellular debris and stored at 4°C for one week. For purification of the protein fraction in the CM, it was separated into nonprotein and protein fractions using the Amicon Ultra 10K (Millipore Sigma).

### Production and Expression of CXCL12

To measure CXCL12 production by the stromal cells, the culture supernatant was collected one day after replacing the media of stromal cells that had reached 80% confluency and was replaced with fresh medium. The concentration of CXCL12 in the culture supernatant was measured using an ELISA (Thermo Fisher Scientific).

### Proliferative Capacity, Cytotoxicity, IL2 Production, and Cytokine Staining

The T cells were labeled with 2.5 μmol/L of CellTrace Violet (Thermo Fisher Scientific) at 37°C for 8 minutes in PBS and stimulated with 40 Gy–irradiated NALM6 cells (E:T ratio = 4:1 or 1:1) for three days. Cell division, phenotype, and the number of live T cells were measured on the FACSCanto II (BD Biosciences). To measure IL2 production by the T cells, the culture supernatants were collected 22–24 hours after the T cells were stimulated with the 40 Gy–irradiated NALM6 cells (E:T ratio = 1:1). The concentration of IL2 in the culture supernatant was measured using an ELISA (eBioscience). To measure cytokine production, the T cells were stimulated with K562-hCD19 cells (E:T ratio = 1:1) for 6 hours, in the presence of brefeldin A (1:1,000 dilution). Intracellular IFNγ, TNFα, and IL2 were stained and analyzed by flow cytometry. To measure cytotoxicity, NALM6 cells were labeled with 2.5 μmol/L of CellTrace Violet at 37°C for 8 minutes in PBS and then T cells were cocultured with the labeled NALM6 cells (E:T ratio = 8:1, 4:1, or 1:1) for 3 hours. Dead NALM6 cells (CD8α^−^ CellTrace Violet^+^PI^+^ cells) were measured using flow cytometry analysis.

### Flow Cytometry

Fluorochrome-conjugated monoclonal/polyclonal anti-human CD8a (300924 or 300912, HIT8a), anti-human CD45RA (304126 or 304152, HI100), anti-human CCR7 (353218 or 353225, G043H7), anti-human CD45RO (304228, UCHL1), anti-human CD27 (356406, M-T271), anti-human CD62 L (304828, DREG-56), anti-human CD25 (302606, BC96), anti-human CD28 (302907, CD28.2), anti-human CD95 (305608, DX2), anti-human PD-1 (562516, EH12.1), anti-human CXCR3 (353707, G025H7), anti-human CXCR4 (306505, 12G5), anti-human CXCR7 (391403, 10D1-J16), anti-human CD19 (302208, HIB19), anti-human CD45 (25–0459–42, HI30), anti-mouse CD45 (103134, 30-F11), anti-human LAG-3 (369305, 11C3C65), anti-human CD39 (12–0399–42, EBIOA1), anti-human TIGIT (17–9500–42, MBSA43), anti-human CD69 (17–0699–42, FN50), anti-human IL2 (25–7029–42, MQ1–17H12), anti-human TNFα (12–7349–82, MAb11), anti-human IFNγ (17–7319–82, 4SB3), anti-human TCF1 (655207, 7F11A10), anti-human phospho-γH2AX (12–9865–42, CR55T33), propidium iodide (421301; PI), and fixable viability Dye eFluor 780 (65–0865–14; FVD) were purchased from eBioscience (BioLegend), BD Biosciences, or Thermo Fisher Scientific.

To measure glucose uptake ability, T cells were incubated with 100 μmol/L 2-NBDG (Invitrogen) at 37°C for 2 hours in glucose-free RPMI1640 medium supplemented with 10% AB serum and then washed twice before flow cytometry. To measure mitochondrial membrane potential, cells were stained with 100 nmol/L of tetramethylrhodamine, methyl ester (TMRM; Thermo Fisher Scientific) at 37°C for 20 minutes in RPMI medium supplemented with 10% AB serum. To measure the total cellular reactive oxygen species (ROS), cells were stained with 1 μmol/L CellROX (Thermo Fisher Scientific) at 37°C for 30 minutes in RPMI medium with 10% AB serum. Intracellular staining was performed using a fixation/permeabilization buffer solution (eBioscience) or an intracellular fixation buffer (eBioscience), according to the manufacturer's instructions. We performed flow cytometry acquisition on a FACSCanto II cytometer (BD Biosciences) and analyzed the data using FlowJo software (Tree Star). Human and mouse cells were sorted using a FACSAria III cell sorter (BD Biosciences).

### Quantitative Real-Time PCR

Total RNA was extracted using the ReliaPrep RNA Miniprep System (Promega) and subsequently reverse transcribed using the High-Capacity cDNA Reverse Transcription Kit (Thermo Fisher Scientific) as described previously (11). PCR analysis was performed using an iCycler iQ multicolor Real-Time PCR Detection System (Bio-Rad Laboratories) and the SsoFast EvaGreen Supermix (Bio-Rad). All primer sets yielded a single product of the correct size. Relative expression levels were normalized to 18SrRNA.

### RNA-Sequencing Analysis

RNA-sequencing (RNA-seq) analysis was performed by GENEWIZ or ImmunoGeneTeqs, Inc.. For RNA isolation, CAR T cells, FC CAR-iT_SCM_ cells, and FF CAR-iT_SCM_ cells were prepared as described previously.

For RNA sequencing by GENEWIZ, total mRNA was isolated using RNeasy Plus Mini Kit (QIAGEN). RNA sequencing by GENEWIZ was performed using Illumina HiSeq 4000 platform, after trimming adapters using Cutadapt and checked for quality using FASTQC, pair-end reads were aligned to human reference genome (hg38) using Hisat2.

For RNA sequencing by ImmunoGeneTeqs, T cells were sorted into Lysis buffer and freezed at −80°C and then we submitted these cells to ImmunoGeneTeqs. RNA sequencing by ImmunoGeneTeqs was performed using Illumina NovaSeq 6000 platform, after trimming adapters using Cutadapt and checked for quality using FASTQC, 50 bp single-end reads were aligned to human RefSeq RNA using bowtie2.

We obtained gene expression data file from GENEWIZ or ImmunoGeneTeqs and further statistical analyses were performed using the R statistical language and environment. Principal component analysis was performed using the prcomp function from the stats package and visualized using the base R graphics. Statistical significance of differentially expressed genes (DEG) was assessed using the edgeR package (version 3.22.1) and visualized using the base R graphics. Gene expression was visualized using the heatmap.2 function from the gplots package. The gene set enrichment analysis (GSEA) was performed using the GSEA software (University of California, San Diego, San Diego CA and the Broad Institute, Cambridge, MA).

### Measurement of Extracellular Acidification Rate and Oxygen Consumption Rate

Oxygen consumption rate (OCR) and the extracellular acidification rate (ECAR) were measured using a XFe24 Extracellular Flux Analyzer (Agilent Technologies). The ECAR and the OCR were measured with the Seahorse XF Cell Mito Stress Test Kit (Agilent Technologies) as described previously (11).

### Adoptive T-Cell Transfer into Humanized Leukemia Mice

The tumor transplantation and therapeutic model was performed according to the procedure reported by Kondo and colleagues (11). Briefly, male NSG mice that were 6 to 10 weeks old were inoculated with 1 × 10^6^ NALM6 leukemia cells via intravenous injection seven days before CAR T-cell transfer. CAR T cells (5 × 10^5^) were adoptively transferred into NALM6-bearing mice. On days 4, 5, 14, and 25 after CAR T-cell transfer, the peripheral blood and spleens from the mice were collected, and the erythrocytes were lysed. The numbers of circulating NALM6 cells (mCD45^−^FVD^−^hCD8α^−^hCD19^+^) and CAR T cells (mCD45^−^FVD^−^hCD45^+^hCD8α^+^Venus^+^) were measured and analyzed by flow cytometry. Animals were closely monitored for signs of graft-versus-host disease (GvHD) and other toxicity, as evidenced by loss of fur, diarrhea, or conjunctivitis, as well as for leukemia-related hind-limb paralysis. When mice reached the humane endpoint in the tumor model, they were sacrificed, as approved by the Animal Ethics Committee of Keio University (Tokyo, Japan).

### Statistical Analyses

Statistical analyses were performed using Student's *t* test, one- or two-way ANOVA with *post hoc* Tukey test, or the Kaplan–Meier method, using GraphPad Prism version 8 software (GraphPad Software). All data are presented as the mean ± SEM.

### Data Availability Statement

The RNA-seq data that support the findings of this study have been deposited in DNA Data Bank of Japan Sequence Read Archive (https://www.ddbj.nig.ac.jp/index.html) with accession numbers DRA012676 and DRA012677.

## Results

### FC-Mediated CAR-iT_SCM_ Cells Show Early Memory Phenotypes, Irrespective of Donors

**FIGURE 1:**
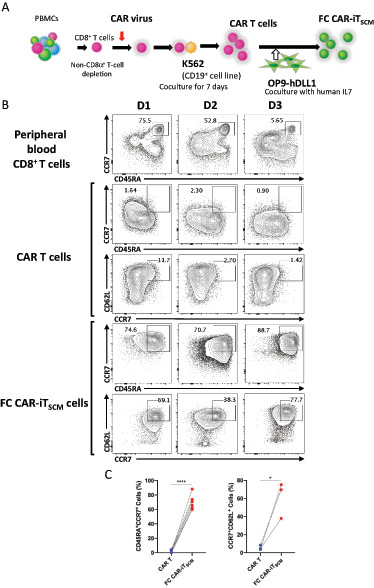
Generation of CAR-iT_SCM_ cells with FCs. **A,** Schematic illustration of the procedures for FC CAR-iT_SCM_ cell induction. **B,** Representative FACS profile of CD45RA and CCR7 expression or CCR7 and CD62L expression in CD8^+^ T cells, CAR T cells, and FC CAR-iT_SCM_ cells from three healthy donors. **C,** The percentage of CD45RA^+^CCR7^+^ cells (left) or CCR7^+^CD62L^+^ cells (right) in CAR T cells and FC CAR-iT_SCM_ cells from 3–6 healthy donors. Data are presented as mean ± SEM. *, *P* < 0.05; ****, *P* < 0.0001; Student's *t* test. Data are representative of at least two independent experiments.

CAR T cells were generated from the whole CD8^+^ T-cell fraction of PBMCs by retroviral CAR cDNA transduction and expansion with CD19-expressing antigen-presenting cells (CD19-K562 cells; Fig. 1A). CAR-iT_SCM_ cells are induced by coculturing activated/exhausted CD8^+^ CAR T cells with OP9-hDLL1 cells (refs. 11, 24, 25; Fig. 1A). We often experienced that the quality of CAR T cells varies depending on donors. As shown in Fig. 1B, after CAR transduction, early memory markers, CCR7, CD62L, and CD45RA, were low but varied depending on donors. However, irrespective of donors, we confirmed that the OP9-hDLL1 FC coculture resulted in the generation of uniform CD45A^+^, CCR7^+^, and CD62L^+^ T_SCM_-like (FC CAR-iT_SCM_) cells (Fig. 1B and C). For example, even though the CAR T cells from donor 3 (D3), who had the fewest naïve cells in PBMCs and CAR T cells, expressed almost no naïve markers, the FC CAR-iT_SCM_ cells expressed higher levels of early memory markers and genes including *CD27*, *CD62L(SELL)*, *TCF7*, *LEF1*, and *IL7R* but lower exhaustion marker genes such as *PDCD1*, *TOX*, *TOX2*, *NR4A*, and *LAG3* (Supplementary Fig. S1A and B). These results indicate that the FC coculture uniforms the phenotypes of CAR T cells as T_SCM_-like cells independent on donors.

### Identification of CXCL12 as a Key Factor for CAR-iT_SCM_ Induction

**FIGURE 2:**
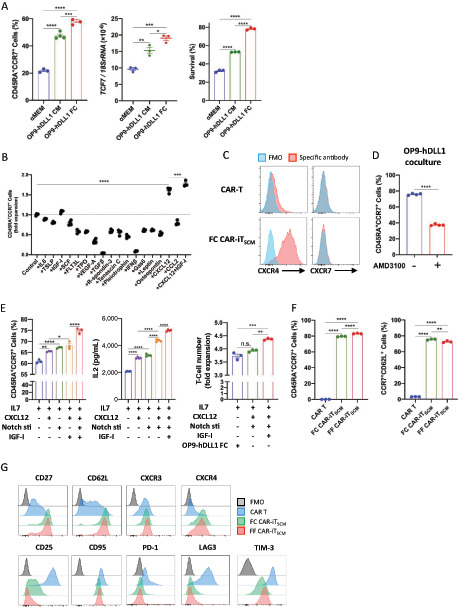
Establishment of FF culture system for CAR-iT_SCM_ induction. **A,** The percentage of CD45RA^+^CCR7^+^ cells, survival, or gene expression of *TCF7* in CAR-iT_SCM_ cells induced by αMEM, OP9-hDLL1 CM, and OP9-hDLL1 FC for 10 days. **B,** Fold expansion of CD45RA^+^CCR7^+^ cells cultured in the presence of the indicated growth factors for 10 days. **C,** CXCR4 and CXCR7 expression on CAR T and FC CAR-iT_SCM_ cells. The blue histograms represent the fluorescent minus one as the controls. **D,** The percentage of CD45RA^+^CCR7^+^ cells in CAR-iT_SCM_ cells induced by OP9-hDLL1 FC for 10 days in the presence of DW or AMD3100 (6 μmol/L). **E,** The percentage of CD45RA^+^CCR7^+^ cells in the CAR-iT_SCM_ cell population that was induced by the indicated conditions for 10 days (left). After CAR-iT_SCM_ induction by the indicated conditions, IL2 release was measured from CAR-iT_SCM_ cells that were stimulated with 40 Gy–irradiated NALM6 cells for 24 hours (middle) or the fold expansion of CAR-iT_SCM_ cells cocultured with 40 Gy–irradiated NALM6 cells for three days was determined (right). **F,** The percentage of CD45RA^+^CCR7^+^ cells or CCR7^+^CD62L^+^ cells in the CAR T and CAR-iT_SCM_ cell populations that were induced by OP9-hDLL1 FCs (FC CAR-iT_SCM_) and the FF culture system (IL7 + IGF-I + CXCL12 + Notch stimulation, FF CAR-iT_SCM_) for 10 days. **G,** The surface marker expression of CD27, CD62L, CXCR3, and CXCR4 as naïve/early memory markers, CD25 and CD95 as activation markers, and PD-1, LAG3, and TIM-3 as exhaustion markers in CAR-T, FC CAR-iT_SCM_, and FF CAR-iT_SCM_ cells. The gray histograms represent the fluorescent minus one as the controls. Data are presented as mean ± SEM. *, *P* < 0.05; **, *P* < 0.01; ***, *P* < 0.001; ****, *P* < 0.0001; n.s., not significant; Student's *t* test (**D**), one-way ANOVA (**A**, **B**, **E**, **F**). Data are representative of at least two independent experiments.

Although OP9-hDLL1 FCs induced CAR-iT_SCM_ cells very efficiently, long-term coculture with mouse FCs limits clinical applications of the method. Therefore, we tried to define the factors from OP9-hDLL1 cells that are involved in CAR-iT_SCM_ induction to establish a new FF iT_SCM_ induction system. It has been reported that the CM used for various FCs promotes T-cell survival, proliferation, and memory differentiation (27–30). Indeed, culturing activated CAR T cells with OP9-hDLL1 CM, in the presence of IL7, partially converted CD45A^−^CCR7^−^–activated T cells into CD45A^+^CCR7^+^ T_SCM_–like cells with increased *TCF7* expression and survival, although all these T_SCM_ abilities are lower than those induced by OP9-hDLL1 FCs (Fig. 2A).

First, we confirmed that a high molecular weight fraction of the OP9-hDLL1 CM promoted the CD45A^+^CCR7^+^ phenotype (Supplementary Fig. S2A). Whereas the NIH 3T3 (fibroblast) CM did not induce the CD45A^+^CCR7^+^ phenotype, CM derived from TSt-4 (stromal) cells promoted the induction of the CD45A^+^CCR7^+^ phenotype more efficiently than OP9 cells (Supplementary Fig. S2B). We hypothesized that secreted proteins from murine stromal cells induced CD45RA^+^CCR7^+^ phenotype. Previous reports have listed various secreted proteins from stromal cells (31–34). We therefore examined the effect of each of these stromal cell–derived secreted proteins, which have homology with human proteins, on CD45A^+^CCR7^+^–naïve marker induction (Fig. 2B). We discovered that CXCL12 [stromal cell–derived factor-1α (SDF-1α)] promoted the CD45A^+^CCR7^+^ phenotype in CAR T cells similar to the CM of OP9-hDLL1 cells. The protein level of CXCL12 in the TSt-4 CM was higher than in the OP9 CM and was undetectable in the NIH 3T3 CM (Supplementary Fig. S2C), which is well correlated with the naïve marker induction potency of TSt-4 and NIH 3T3 CMs.

Among the CXCL12 receptors, CXCR4, but not CXCR7, was expressed in CAR T cells and further increased during coculture with OP9-hDLL1 cells (Fig. 2C). To confirm that the CXCR4 signal is involved in the induction of CAR-iT_SCM_ cells, the effect of a CXCR4 inhibitor (AMD3100) on CAR-iT_SCM_ induction was examined. Treatment with AMD3100 markedly reduced the efficiency of CAR-iT_SCM_ induction by the OP9-hDLL1 coculture (Fig. 2D). AMD3100 also reduced CD45A^+^CCR7^+^ induction by the CMs of OP9, OP9-hDLL1, and TSt-4 cells (Supplementary Fig. S2D). These data indicate that the CXCL12–CXCR4 pathway is important for CAR-iT_SCM_ formation.

### Optimization of FF CAR-iT_SCM_ Induction

The efficiency of CM for the induction of the T_SCM_ phenotype did not reach the level of the coculture with OP9-hDLL1 FCs as shown in Fig. 2A, suggesting that additional factors are necessary for full iT_SCM_ induction. Thus, we examined the effect of the Notch ligand and IGF-I signals. We previously reported that Notch ligand/signals enhanced iT_SCM_ formation (11, 24, 25). IGF-I, which is known to be derived from stromal cells as mentioned before, has often been used to replace serum and is known to be involved in T-cell differentiation, survival, and proliferation (34).

The plate coated with hDLL1-Fc protein increased expression of the Notch target genes *HEY1* and *HES1* (Supplementary Fig. S3A). Several reports have shown that the combination of Notch ligand and FN elicit synergistic effects for T-cell development and maturation (35, 36). We examined the synergistic effects of FN on Notch signaling, and found that a FN coating on the plate further enhanced the expression of *HEY1* and *HES1* (Supplementary Fig. S3A). IGF-I further increased CD45A^+^CCR7^+^ fraction, IL2 production, and cell proliferation (Fig. 2E). We demonstrated that FF condition, that is, IL7, CXCL12, Notch, and IGF-I cooperatively increased CD45A, CCR7, and CD62L–naïve marker expression to the comparable levels in FC CAR-iT_SCM_ cells (Fig. 2F; Supplementary Fig. S3B), which was also confirmed in additional two independent donors (Supplementary Fig. S3C). Furthermore, FF CAR-iT_SCM_ cells expressed other T_SCM_/memory markers including CD27, CD28, CD62L, CXCR3, and CXCR4 and reduced checkpoint molecules including PD-1, LAG3, and TIM-3, which was similar to FC CAR-iT_SCM_ cells (Fig. 2G).

### Gene Expression Profiles of FF CAR-iT_SCM_ Cells

**FIGURE 3:**
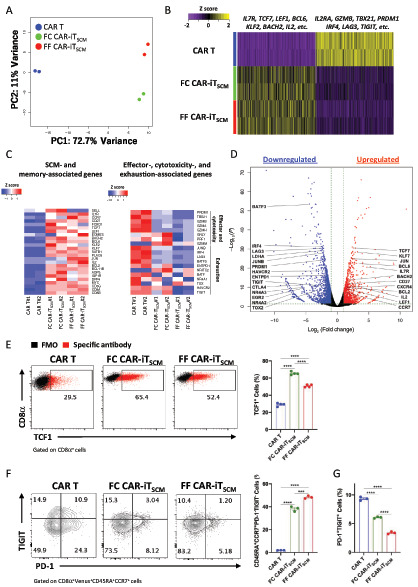
Gene expression analysis among CAR T, FC CAR-iT_SCM_, and FF CAR-iT_SCM_ cells. CAR T cells were expanded for 12–14 days and FC CAR-iT_SCM_ cells were induced by coculturing CAR T cells and FCs for 10 days in the presence of IL7, as shown in Fig. 1A. FF CAR-iT_SCM_ cells were induced by resting CAR T cells in the FF culture system (IL7 + IGF-I + CXCL12 + Notch stimulation) for 10 days. **A,** PC analysis of RNA-sequencing data for CAR T, FC CAR-iT_SCM_, and FF CAR-iT_SCM_ cells. PC1 separates CAR T cells from both FC CAR-iT_SCM_ and FF CAR-iT_SCM_ cells. **B,** Gene expression of PC1-driving 1,000 genes in CAR T, FC CAR-iT_SCM_, and FF CAR-iT_SCM_ cells. **C,** Heat map of selected genes associated with SCM or memory, effector, cytotoxicity, and exhaustion in CAR T, FC CAR-iT_SCM_, and FF CAR-iT_SCM_ cells. **D,** Volcano plot representation of DEGs between CAR T cells and FF CAR-iT_SCM_ cells with a log_2_ fold change >1 and *P*_adj_ < 0.05. **E,** The percentage of TCF1^+^ cells in the CAR T, FC CAR-iT_SCM_, and FF CAR-iT_SCM_ cell populations. The black plot represents the fluorescent minus one as the controls. **F,** The percentage of CD45RA^+^CCR7^+^PD-1^−^TIGIT^−^ cells in the CAR-T, FC CAR-iT_SCM_, and FF CAR-iT_SCM_ cell populations. **G,** The percentage of PD-1^+^TIGIT^+^ cells in the CAR-T, FC CAR-iT_SCM_, and FF CAR-iT_SCM_ cell populations. Data are presented as mean ± SEM. ***, *P* < 0.001; ****, *P* < 0.0001; one-way ANOVA (**E-G**). RNA-sequencing analysis was performed by ImmunoGeneTeqs, Inc. Data are representative of at least two independent experiments.

To confirm the genetic characteristics of the FF CAR-iT_SCM_ cells, RNA-sequencing data of CAR T cells, FC CAR-iT_SCM_ cells, and FF CAR-iT_SCM_ cells from two independent donors were compared. Principal component (PC) analysis revealed that FC CAR-iT_SCM_ and FF CAR-iT_SCM_ cells have similar characteristics compared with CAR T cells in PC1, although they are not completely identical in PC2 (Fig. 3A). We suspected that PC1 separated cells based on their characteristics to T_SCM_ cells; thus, we extracted 1,000 genes that are highly involved in the definition of PC1 (Fig. 3B). Enriched genes in PC1 of FC and FF CAR-iT_SCM_ cells included early memory genes such as *TCF7*, *LEF1*, *BACH2*, *IL7R*, *KLF2*, *BCL6*, and *IL2*, which are important for T_CM_ and T_SCM_ characteristics. On the other hand, genes more enriched in CAR T cells were effector- and exhaustion-related genes (10, 37–39), such as *IL2RA*, *GZMB*, *TBX21*, *PRDM1*, *IRF4*, *TIGIT*, and *LAG3* (Fig. 3B). This was further confirmed by generating a volcano plot of from an independent donor and the selected genes (Fig. 3C). DEGs between CAR T cells and FF CAR-iT_SCM_ cells were compared and the analysis revealed that FF CAR-iT_SCM_ cells express more SCM and memory-associated genes and less exhaustion-associated genes compared with CAR T cells (Fig. 3D), which was very similar to that of FC CAR-iT_SCM_ cells (see Supplementary Fig. S1B). Quantitative PCR analysis also revealed that memory-related genes (10, 11) including *TCF7*, *LEF1*, *EOMES*, *BACH2*, *BCL6*, and *KLF2* were upregulated, while exhaustion-related transcription factors (10, 37–39), such as *NR4A1*, *NR4A2*, *BATF*, *IRF4*, *PRDM1*, *TBX21*, and *TOX*, were reduced in both FC and FF CAR-iT_SCM_ cells (Supplementary Fig. S4). Protein levels of TCF1, a SCM marker, were higher in FC and FF CAR-iT_SCM_ cells compared with conventional CAR T cells (Fig. 3E). Galletti and colleagues reported T_SCM_, T_CM_, and T_PEX_ marker genes (38). Gene set enrichment analysis (GSEA) revealed that FF CAR-iT_SCM_ cells show most closest to the T_SCM_-like gene expression profile, not T_CM_ or T_PEX_ (Supplementary Fig. S5A).

Recent studies have shown that CD39^−^CD69^−^ T cells have T_SCM_ properties in tumor-infiltrating lymphocyte therapy (40), and Galletti and colleagues (38) classified CCR7^+^ cells of CD8^+^ T cells in peripheral blood as follows:

T_SCM_: CD45RA^+^CD45RO^−^CCR7^+^(GZMK^−^)PD-1^−^TIGIT^−^
T_CM_: CD45RA^−^CD45RO^+^CCR7^+^(GZMK^−^)PD-1^−^TIGIT^−^
T_PEX_: CCR7^+^(GZMK^+^)PD-1^+^TIGIT^+^

Consistently, in our human CAR-T system, the fractions of CD69^−^CD39^−^ cells as well as CD45RA^+^CCR7^+^PD-1^−^TIGIT^−^ cells were increased and PD-1^+^TIGIT^+^ cells were decreased in FC and FF CAR-iT_SCM_ cells compared with CAR T cells (Supplementary Fig. S5B and Fig. 3F and G). Interestingly, FF CAR-iT_SCM_ cells were even less exhausted than FC CAR-iT_SCM_ cells. These data further supported that the FF CAR-iT_SCM_ cells are very close to T_SCM_ cells.

### OXPHOS-Dominant Metabolic State of FF CAR-iT_SCM_ Cells

**FIGURE 4:**
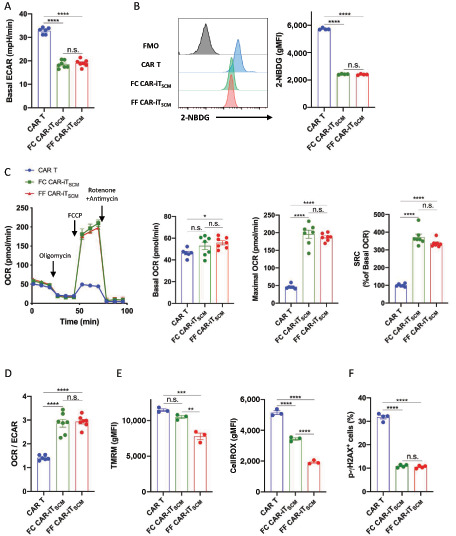
Metabolic state analysis of FF CAR-iT_SCM_ cells. CAR T cells were expanded for 12–14 days and FC CAR-iT_SCM_ cells were induced by coculturing CAR T cells and FCs for 10 days in the presence of IL7, as shown in Fig. 1A. FF CAR-iT_SCM_ cells were induced by resting CAR T cells in the FF culture system (IL7 + IGF-I + CXCL12 + Notch stimulation) for 10 days. **A,** ECAR measurement for CAR T, FC CAR-iT_SCM_, and FF CAR-iT_SCM_ cells. **B,** FACS analysis of 2-NBDG uptake in CAR T, FC CAR-iT_SCM_, and FF CAR-iT_SCM_ cells. The gray histograms represent the fluorescent minus one as the controls. **C,** OCR measurement for CAR T, FC CAR-iT_SCM_, and FF CAR-iT_SCM_ cells. **D,** OCR/ECAR ratio in CAR-T, FC CAR-iT_SCM_, and FF CAR-iT_SCM_ cells. **E,** FACS analysis of the mitochondrial membrane potential using TMRM (left) or cellular ROS using CellROX (right) in CAR T, FC CAR-iT_SCM_, and FF CAR-iT_SCM_ cells. **F,** The percentage of phospho-γH2AX^+^ (p-γH2AX^+^) cells in CAR T, FC CAR-iT_SCM_, and FF CAR-iT_SCM_ cells. Data are presented as mean ± SEM. *, *P* < 0.05; **, *P* < 0.01; ***, *P* < 0.001; ****, *P* < 0.01; n.s., not significant; one-way ANOVA. Data are representative of at least two independent experiments.

Less differentiated early memory T cells are in an OXPHOS-dominant and glycolysis-limited metabolic state, which is known to enhance survival and persistence; therefore, T_SCM_ cells can produce more effector T cells against tumors (14, 22). The GSEA revealed that the glycolysis-related genes were more enriched in CAR T cells compared with both FC and FF CAR-iT_SCM_ cells (Supplementary Fig. S6A and B). In fact, FC CAR-iT_SCM_ cells and FF CAR-iT_SCM_ cells showed a reduced ECAR (Fig. 4A) and lower glucose uptake ability compared with CAR T cells (Fig. 4B). On the other hand, although we did not observe apparent differences in the OXPHOS-related gene expression, both FC and FF CAR-iT_SCM_ cells showed much higher oxygen consumption rate (OCR) compared with CAR T cells (Fig. 4C). The basal OCR/ECAR ratio was higher in FC and FF CAR-iT_SCM_ cells compared with CAR T cells, indicating that both FC and FF CAR-iT_SCM_ cells highly preferred OXPHOS to glycolysis (Fig. 4D).

T_CM_ and T_SCM_ cells were previously found to possess a lower mitochondrial membrane potential, which resulted in a decrease in oxidative stress and DNA damage (41). Consistent with the previous report, the low mitochondrial membrane potential fraction of CAR T cells showed more of a memory cell phenotype, while the high mitochondrial membrane potential fraction contained more of the CD45RA^+^CCR7^−^ (T_EMRA_) phenotypes (Supplementary Fig. S6C–S6E). As expected, FF CAR-iT_SCM_ cells had a lower mitochondrial membrane potential and a lower level of ROS accumulation than FC CAR-iT_SCM_ cells (Fig. 4E and Supplementary Fig. S7 for FACS profile). Compared with CAR T cells, FC and FF CAR-iT_SCM_ cells had fewer phospho-γH2AX–positive cells, which is an indicator of DNA damage (Fig. 4F and Supplementary Fig. S7 for FACS profile). All these metabolic features also supported our proposal that FF CAR-iT_SCM_ cells have stronger T_SCM_ properties.

### FF CAR-iT_SCM_ Cells have a Longer Persistence and Higher Antitumor Potential than CAR T Cells *In Vitro*

**FIGURE 5:**
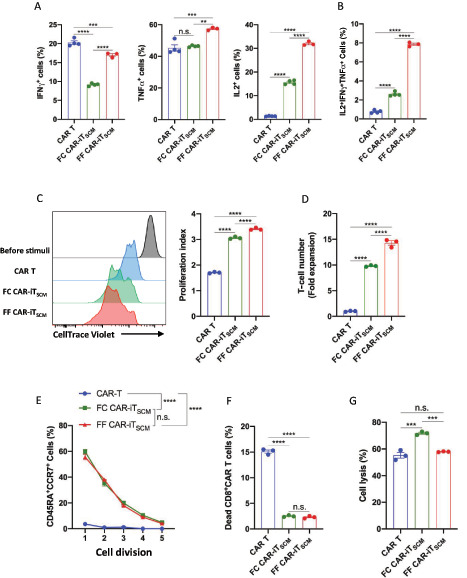
Characterization of FF CAR-iT_SCM_ cells *in vitro*. CAR T cells were expanded for 12–14 days and FC CAR-iT_SCM_ cells were induced by coculturing CAR T cells and FCs for 10 days in the presence of IL7, as shown in Fig. 1A. FF CAR-iT_SCM_ cells were induced by resting CAR T cells in the FF culture system (IL7 + IGF-I + CXCL12 + Notch stimulation) for 10 days. FACS analysis of IFNγ^+^, TNFα^+^, IL2^+^ (**A**), and IFNγ^+^TNFα^+^IL2^+^ (**B**) cells in CAR T, FC CAR-iT_SCM_, and FF CAR-iT_SCM_ cells 6 hours after stimulation with K562-hCD19 cells. **C,** FACS analysis of CellTrace Violet dilution of CellTrace Violet-labeled cells three days after coculture with 40 Gy–irradiated NALM6 cells. **D,** Fold expansion of CAR T, FC CAR-iT_SCM_, and FF CAR-iT_SCM_ cells three days after coculture with 40 Gy—irradiated NALM6 cells. **E,** The percentage of CD45RA^+^CCR7^+^ cells in CellTrace Violet–diluted CAR T, FC CAR-iT_SCM_, and FF CAR-iT_SCM_ cells. Dead CAR T, FC CAR-iT_SCM_, and FF CAR-iT_SCM_ cells (**F**) 3 hours after stimulation with NALM6 cells or the percentage of dead CellTrace Violet–labeled NALM6 cells (**G**) by coculture with CAR T, FC CAR-iT_SCM_, and FF CAR-iT_SCM_ cells for three hours. Data are presented as mean ± SEM. **, *P* < 0.01; ***, *P* < 0.001; ****, *P* < 0.0001; n.s., not significant; one-way ANOVA (**A-D**, **F**, and **G**) or two-way ANOVA (**E**). Data are representative of at least two independent experiments.

Next, we compared persistence and the antitumor potential among FC CAR-iT_SCM_ cells, FF CAR-iT_SCM_ cells, and conventional CAR T cells *in vitro*. After restimulation with K562-hCD19 cells, IFNγ, TNFα, and IL2-positive cells were measured by flow cytometry. Both FC and FF CAR-iT_SCM_ cells showed decreased IFNγ production and increased IL2 production compared with CAR T cells (Fig. 5A). Interestingly, FF CAR-iT_SCM_ cells showed higher IFNγ, TNFα, and IL2 levels compared with FC CAR-iT_SCM_ cells (Fig. 5A). As a result, FF CAR-iT_SCM_ cells had a significantly higher multipotential IL2^+^IFNγ^+^TNFα^+^ fraction than both CAR T cells and FC CAR-iT_SCM_ cells (Fig. 5B).

We next investigated the proliferation potential of FF CAR-iT_SCM_ cells in response to human leukemic CD19^+^ NALM6 (Fig. 5C and D). FC and FF CAR-iT_SCM_ cells proliferated faster than CAR T cells, and the total number of FC and FF CAR-iT_SCM_ cells was much higher than that of CAR T cells. Importantly, FF CAR-iT_SCM_ cells showed superior proliferative ability compared with FC CAR-iT_SCM_ cells *in vitro* (Fig. 5C and D). Furthermore, FC and FF CAR-iT_SCM_ cells partially retained the CD45A^+^CCR7^+^–naïve phenotype after serial cell division, which suggests that FC and FF CAR-iT_SCM_ cells have the potential to self-renew (Fig. 5E). In addition, FC and FF CAR-iT_SCM_ cells were more resistant to cell death than CAR T cells (Fig. 5F). *In vitro* cytotoxic activity of FF CAR-iT_SCM_ cells was slightly lower than that of FC CAR-iT_SCM_ cells; however, it was not significantly different to that of CAR T cells (Fig. 5G). These results indicate that FF CAR-iT_SCM_ cells exhibit multifunctionality, excellent proliferative ability, an ability to self-renew, and are resistant to cell death.

### FF CAR-iT_SCM_ Cells Possess Strong Antitumor Potential *In Vivo*

**FIGURE 6:**
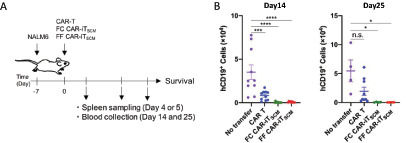

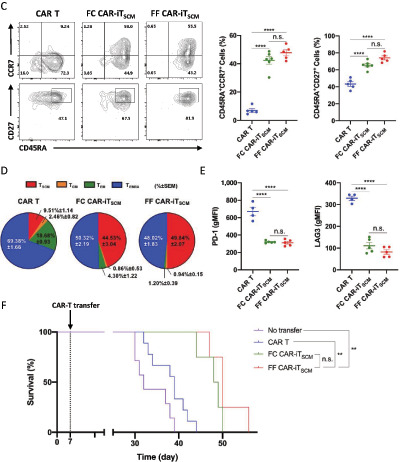
*In vivo* antitumor effects of FF CAR-iT_SCM_ cells. CAR T cells were expanded for 12–14 days and FC CAR-iT_SCM_ cells were induced by coculturing CAR T cells and FCs for 10 days in the presence of IL7, as shown in Fig. 1A. FF CAR-iT_SCM_ cells were induced by resting CAR T cells in the FF culture system (IL7 + IGF-I + CXCL12 + Notch stimulation) for 10 days. **A,** Scheme of CAR T-cell transfer into humanized leukemia mice. **B,** The number of circulating NALM6 cells in the peripheral blood in the indicated mice on day 14 after CAR T-cell transfer (*n* = 4 for no transfer, *n* = 9 for CAR T-cell transfer, *n* = 4 for FC CAR-iT_SCM_ transfer, *n* = 4 for FF CAR-iT_SCM_ transfer) or day 25 (*n* = 6 for no transfer, *n* = 5 for CAR T-cell transfer, *n* = 9 for FC CAR-iT_SCM_ transfer, *n* = 8 for FF CAR-iT_SCM_ transfer). **C,** The percentage of CD45RA^+^CCR7^+^ cells (top) and CD45RA^+^CD27^+^ cells (bottom) in the spleen of mice that received a transfer of CAR T, FC CAR-iT_SCM_, or FF CAR-iT_SCM_ cells four days after CAR T-cell transfer. **D,** The percentage of each subset of T cells including T_SCM_ (CD45RA^+^CCR7^+^), T_CM_ (CD45RA^−^CCR7^+^), T_EM_ (CD45RA^−^CCR7^−^), and T_TEMRA_ (CD45RA^+^CCR7^−^) cells in the spleen of mice that received a transfer of CAR-T, FC CAR-iT_SCM_, or FF CAR-iT_SCM_ cells four days after CAR T-cell transfer. **E,** PD-1 and LAG-3 expression on CAR T, FC CAR-iT_SCM_, and FF CAR-iT_SCM_ cells in the spleen of NALM6-bearing mice five days after CAR T-cell transfer. **F,** Survival rates of NALM6-bearing mice (*n* = 7 for no transfer, *n* = 9 for CAR T-cell transfer, *n* = 4 for FC CAR-iT_SCM_ transfer, *n* = 4 for FF CAR-iT_SCM_ transfer). Data are presented as mean ± SEM. *, *P* < 0.05; **, *P* < 0.01; ***, *P* < 0.001; ****, *P* < 0.0001; n.s., not significant; one-way ANOVA (**B-C** and **E**); the Kaplan–Meier estimator (**F**). Data are representative of at least two independent experiments.

To verify the antitumor effect of FF CAR-iT_SCM_ cells *in vivo*, CAR T cells, FC CAR-iT_SCM_ cells, and FF CAR-iT_SCM_ cells were transferred into NSG mice bearing NALM6 cells (Fig. 6A). Fourteen days after the CAR T-cell transfer, the number of leukemia cells was decreased; however, a large number of leukemia cells was detected in mice on day 25 after CAR-T transfer, whereas much fewer leukemia cells were detected even 25 days after FC and FF CAR-iT_SCM_ cells were transferred into mice (Fig. 6B).

A large fraction of CD8^+^ T cells retained the T_SCM_ phenotype (CD45RA^+^CCR7^+^ and CD45RA^+^CD27^+^) in mice where FC CAR-iT_SCM_ or FF CAR-iT_SCM_ cells were transferred four days posttransfer (Fig. 6C and D). On the other hand, transferred conventional CAR T cells displayed low T_SCM_ phenotype and a high terminally differentiated phenotype (CD45RA^+^CCR7^−^ cells; Fig. 6C and D). In addition, FC and FF CAR-iT_SCM_ cells retained low expression levels of the T-cell exhaustion markers PD-1 and LAG3 in five days posttransfer (Fig. 6E).

Finally, we compared survival of tumor-bearing NSG mice treated with or without various CAR T cells. There were no signs of GvHD, suggesting that the mice died from leukemia. Mice that received FC CAR-iT_SCM_ or FF CAR-iT_SCM_ cells survived longer compared with mice that received CAR T cells (Fig. 6F). FF CAR-iT_SCM_ cells seems to be slightly more effective than FF CAR-iT_SCM_ cells. These results indicate that FF CAR-iT_SCM_ cells exhibit a potent antitumor effect over the long term and are able to maintain the T_SCM_ phenotype *in vivo*.

## Discussion

CAR T-cell therapy is anticipated to become a very effective immunotherapy strategy against tumors. However, some patients who have received CAR T-cell therapy relapse or do not respond to it, due to loss of transfused CAR T cells within a few months after transfer. In addition, treatment of solid tumors with CAR T-cell therapy has not been successful. Limited persistence of transfused CAR T cells along with the rapid exhaustion of these cells during preparation and within the body is one of the challenges to be overcome with CAR T-cell therapy. The molecular mechanism of T-cell exhaustion has been extensively studied recently, and the transcription factors NR4A and TOX have been directly implicated in exhaustion (37, 42–44). However, the exact mechanism of T-cell exhaustion is not yet fully understood, and the methods that revert the exhaustion state to more early memory stages has not been established at all.

We previously reported that SCM-like CAR T cells can be induced by coculturing activated and expanded CAR T cells with Notch ligand–expressing feeder cells (OP9-hDLL1 cells; ref. 11). In this study, we successfully induced FF CAR-iT_SCM_ cells by resting CAR T cells under the conditions of IL7, IGF-I, CXCL12, and Notch stimulation. A whole gene expression analysis demonstrated that FC CAR-iT_SCM_ cells and FF CAR-iT_SCM_ cells are very similar, if not completely identical. We also showed that marker gene expression profiles of FC CAR-iT_SCM_ cells and FF CAR-iT_SCM_ cells are very similar to those of actual T_SCM_ cells present in human peripheral blood (38). Furthermore, the FF system may be more effective than the FC system, because FF CAR-iT_SCM_ cells exhibited significantly higher IL2 production and exhibited superior proliferative capacity than FC CAR-iT_SCM_ cells. Unlike other reported methods, which generate T_SCM_ cells from naïve T cells directly, our FF system can be initiated from previously activated CAR T cells and can increase the cell number. Our method for generating iT_SCM_ cells from activated CAR T cells using the FC-free system could be advantageous for clinical applications.

We suspected that IL7, IGF-I, CXCL12, and Notch ligand transmit signals important for the induction of naïve phenotypes and for cell survival, while resting without TCR signals could also be important for the induction of naïve/T_SCM_ phenotypes. This idea is consistent with a recent report showing that diminishing the CAR tonic signaling for a certain period of time restores the tumor killing activity of CAR T cells (45). However, our method required more than ten days to induce iT_SCM_ cells, this report showed that resting the cells for only four days was sufficient to suppress the expression of exhaustion-related genes, including *PD-1* and increased the expression of memory-related genes. These differences may be due to type of CAR constructs; they used anti-GD2 CAR, which produces strong tonic signals. This article also showed that the low-specificity tyrosine kinase inhibitor dasatinib could be used to rest CAR T cells *in vivo*. Inhibiting tyrosine kinases could be an effective approach to rest CAR-T cells; however, this probably reduces the overall number of T cells. On the other hand, our IL7+CXCL12+IGF-I+Notch stimulation *in vitro* recovery method suppressed cell death and increased T-cell number. This could be advantageous to expand T cells maintaining T_SCM_ phenotypes.

Recent reports have shown that c-Jun overexpression prevents T-cell exhaustion by decreasing and/or displacing an AP-1 inhibitor, such as the BATF, BATF3, and IRF4 from chromatin; furthermore, c-Jun overexpression increases the early memory phenotypes, proliferation, and OXPHOS, which improves T-cell antitumor functions (39, 46). Consistently, FF CAR-iT_SCM_ cells upregulated *JUN* expression and downregulated the expression levels of *BATF*, *BATF3*, *IRF4*, and *JUNB* compared with CAR T cells. In fact, IL2 production and T-cell proliferation were significantly higher in FF CAR-iT_SCM_ cells, and they were less prone to exhaustion *in vivo*. These data suggest that one mechanism to enhance FF CAR-iT_SCM_ cells may be due to the increase the expression of *JUN*.

We have shown that CXCL12 from stromal cells could be a key factor involved in the induction of T_SCM_ features. CXCL12 has been shown to play important roles in the self-renewal of hematopoietic stem cells. CXCR4 is also expressed in memory T cells, and CXCL12 may be important for homeostatic self-renewal of memory T cells in the bone marrow *in vivo* (47, 48).

In conclusion, our FF CAR-iT_SCM_ induction system is valuable for the generation of long lived and more potent antitumor CAR T cells.

## Supplementary Material

Supplementary Figure 1 The surface marker (A) and gene expression profiles (B) which is associated with stem cell memory and exhaustion.

Supplementary Figure 2 CXCL12 in the OP9-hDLL1 CM promotes CAR-iTSCM formation.

Supplementary Figure 3 Schematic illustration of the feeder cell-free culture.

Supplementary Figure 4 Gene expression profiles of FF CAR-iTSCM cells as measured by quantitative PCR.

Supplementary Figure 5 Stem cell memory gene profile and marker expression.

Supplementary Figure 6 Metabolic state analysis of FF CAR-iTSCM cells.

Supplementary Figure 7 Representative FACS profile of the mitochondrial membrane potential, cellular ROS, and DNA damage in FF CAR-iTSCM cells.
