# Supplementary material for: Rejuvenating Effector/Exhausted CAR T Cells to Stem Cell Memory–Like CAR T Cells By Resting Them in the Presence of CXCL12 and the NOTCH Ligand
Source: Cancer Res Commun. 2021 Oct 19;1(1):41–55. doi: 10.1158/2767-9764.CRC-21-0034 (PMC9973402; doi:10.1158/2767-9764.CRC-21-0034)
Supplement: Supplementary Figure 1 — The surface marker (A) and gene expression profiles (B) which is associated with stem cell memory and exhaustion. [file crc-21-0034-s01.pdf]

Supplementary Figure 1

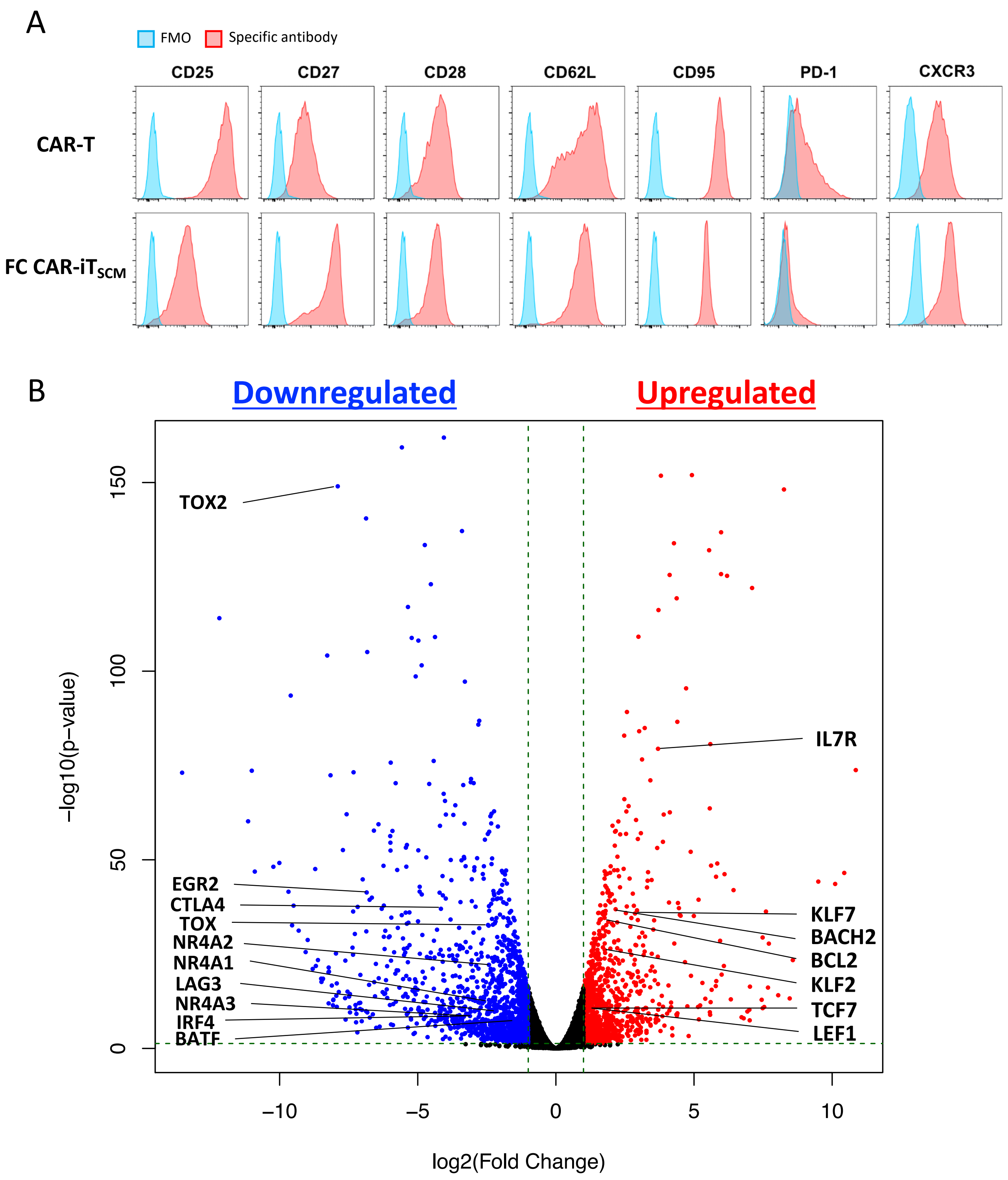

Supplementary Figure 1. The surface marker (A) and gene expression profiles (B) which is associated with stem cell memory and exhaustion.

(**A**) Surface marker and gene expression of CAR-T cells and FC CAR-iT$_{SCM}$ cells from CD8$^+$ T cells of donor D3. The blue histograms represent the fluorescent minus one as the controls. (**B**) Volcano plot representation of differentially expressed genes between CAR-T cells and FC CAR-iT$_{SCM}$ cells with a log2 fold change >1 and adjusted $P < 0.05$.
