# Supplementary material for: Rejuvenating Effector/Exhausted CAR T Cells to Stem Cell Memory–Like CAR T Cells By Resting Them in the Presence of CXCL12 and the NOTCH Ligand
Source: Cancer Res Commun. 2021 Oct 19;1(1):41–55. doi: 10.1158/2767-9764.CRC-21-0034 (PMC9973402; doi:10.1158/2767-9764.CRC-21-0034)
Supplement: Supplementary Figure 2 — CXCL12 in the OP9-hDLL1 CM promotes CAR-iTSCM formation. [file crc-21-0034-s02.pdf]

Supplementary Figure 2

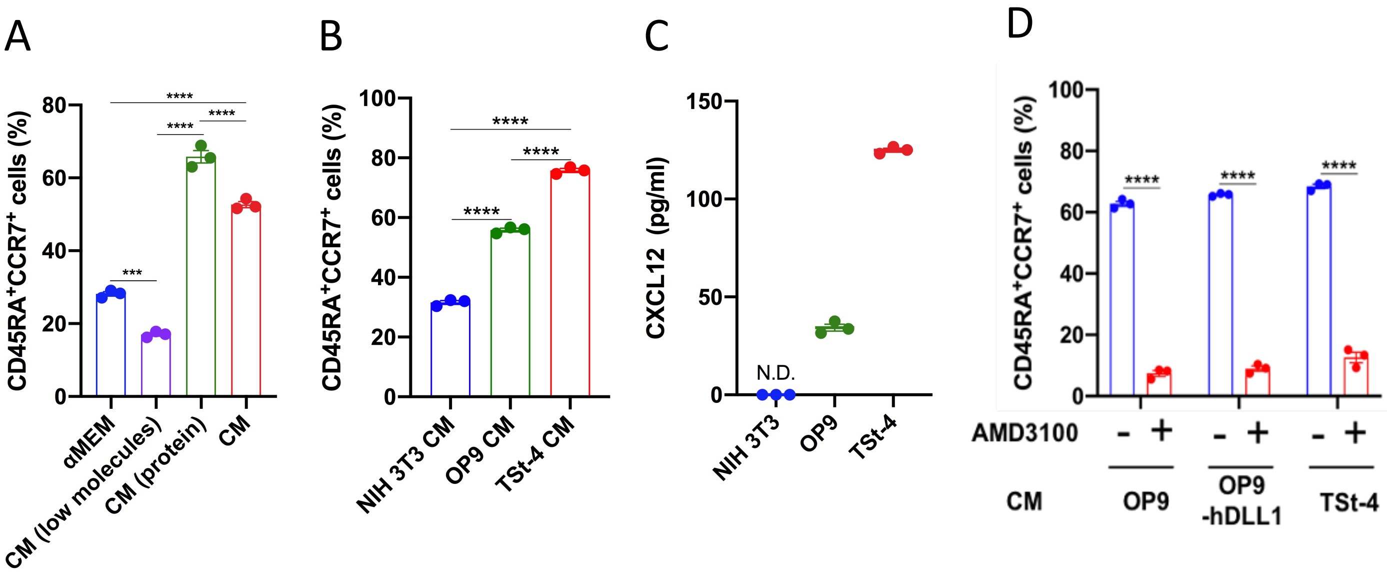

Supplementary Figure 2. CXCL12 in the OP9-hDLL1 CM promotes CAR-iT$_{SCM}$ formation
(**A**) The percentage of CD45RA$^+$CCR7$^+$ cells in CAR-iT$_{SCM}$ cells induced by αMEM, low molecules or protein fractions of OP9-hDLL1 CM. The low molecules or protein fractions of OP9-hDLL1 CM were prepared by ultrafiltration. (**B**) The percentage of CD45RA$^+$CCR7$^+$ cells in CAR-T cells cultured in CMs from OP9 or Tst-4 stromal cells or NIH 3T3 fibroblasts cultured in the presence of IL-7 for 10 days. (**C**) The concentration of CXCL12 measured by ELISA in the CMs from OP9 or TSt-4 stromal cells or NIH 3T3. (**D**) The percentage of CD45RA$^+$CCR7$^+$ cells in CAR-iT$_{SCM}$ cells induced by stromal cells CM in the presence of DW or AMD3100. Data are presented as mean ± SEM. ***, $p < 0.001$; ****, $p < 0.0001$; N.D., not detected; one-way ANOVA (**A-C**) or two-way ANOVA (**D**). Data are representative of at least two independent experiments.
