# Supplementary material for: Rejuvenating Effector/Exhausted CAR T Cells to Stem Cell Memory–Like CAR T Cells By Resting Them in the Presence of CXCL12 and the NOTCH Ligand
Source: Cancer Res Commun. 2021 Oct 19;1(1):41–55. doi: 10.1158/2767-9764.CRC-21-0034 (PMC9973402; doi:10.1158/2767-9764.CRC-21-0034)
Supplement: Supplementary Figure 3 — Schematic illustration of the feeder cell-free culture. [file crc-21-0034-s03.pdf]

Supplementary Figure 3

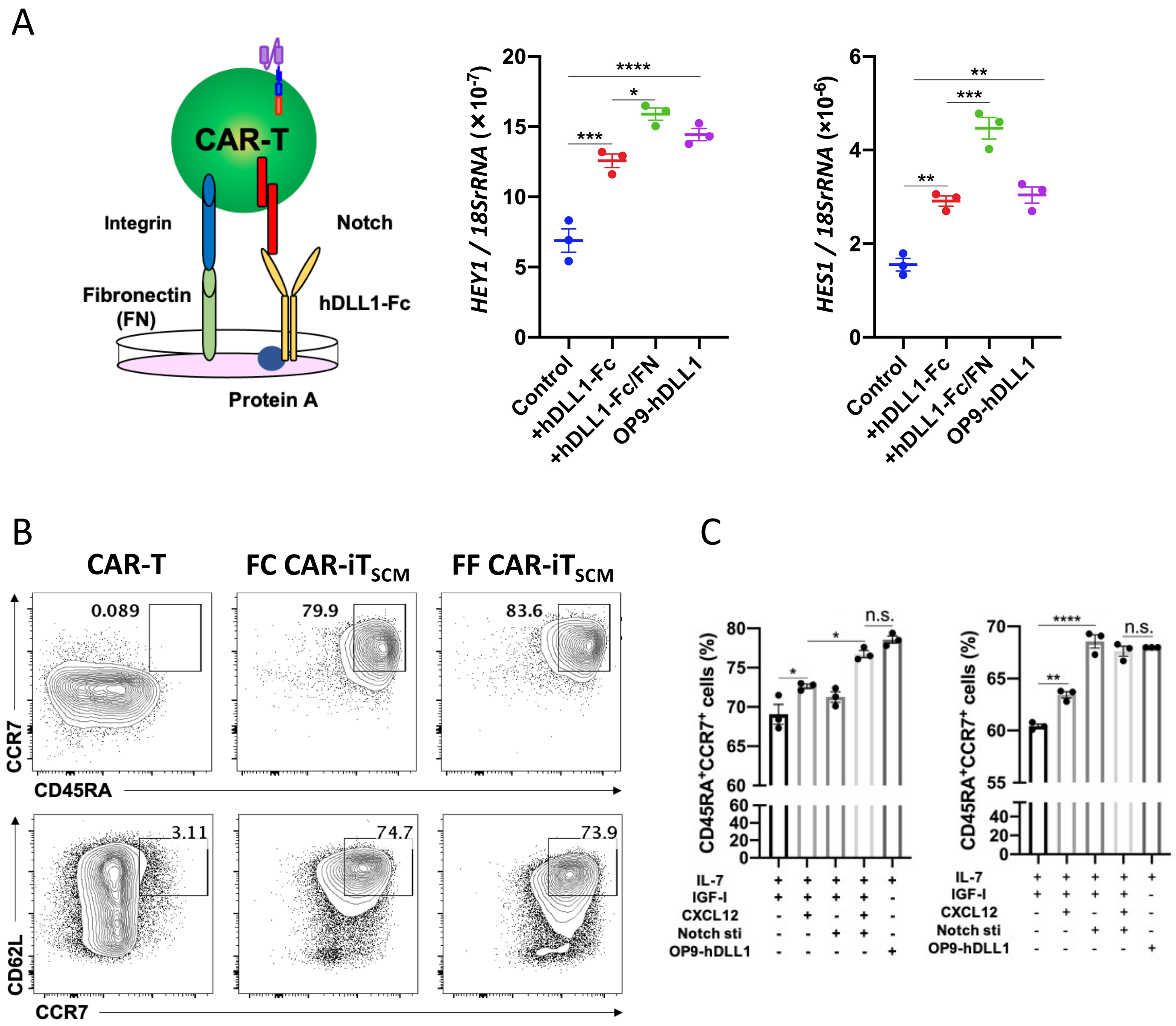

Supplementary Figure 3. Schematic illustration of the feeder cell-free culture

(**A**) To introduce Notch signaling into activated CD8^{+} CAR-T cells, the plate for the feeder-free system was coated with protein A and fibronectin and was subsequently coated with hDLL1-Fc. The expression of the genes *HEY1* and *HES1*, as Notch target genes, in CAR-T cells stimulated with the control, hDLL1-Fc in the presence of fibronectin, and OP9-hDLL1 feeder cells for six hours. (**B**) Representative FACS profile of CD45RA and CCR7 expression or CCR7 and CD62L expression in CAR-T cells, FC CAR-iT$_{SCM}$ cells, and FF CAR-iT$_{SCM}$ cells. (**C**) The percentage of CD45RA^{+}CCR7^{+} cells in the CAR-iT$_{SCM}$ cell population that was induced by the indicated feeder-free conditions from two healthy donors. Data are presented as mean ± SEM. *, $p < 0.05$; **, $p < 0.01$; ***, $p < 0.001$; ****, $p < 0.0001$; n.s., not significant; one-way ANOVA. Data are representative of at least two independent experiments.
