# Supplementary material for: Rejuvenating Effector/Exhausted CAR T Cells to Stem Cell Memory–Like CAR T Cells By Resting Them in the Presence of CXCL12 and the NOTCH Ligand
Source: Cancer Res Commun. 2021 Oct 19;1(1):41–55. doi: 10.1158/2767-9764.CRC-21-0034 (PMC9973402; doi:10.1158/2767-9764.CRC-21-0034)
Supplement: Supplementary Figure 4 — Gene expression profiles of FF CAR-iTSCM cells as measured by quantitative PCR. [file crc-21-0034-s04.pdf]

Supplementary Figure 4

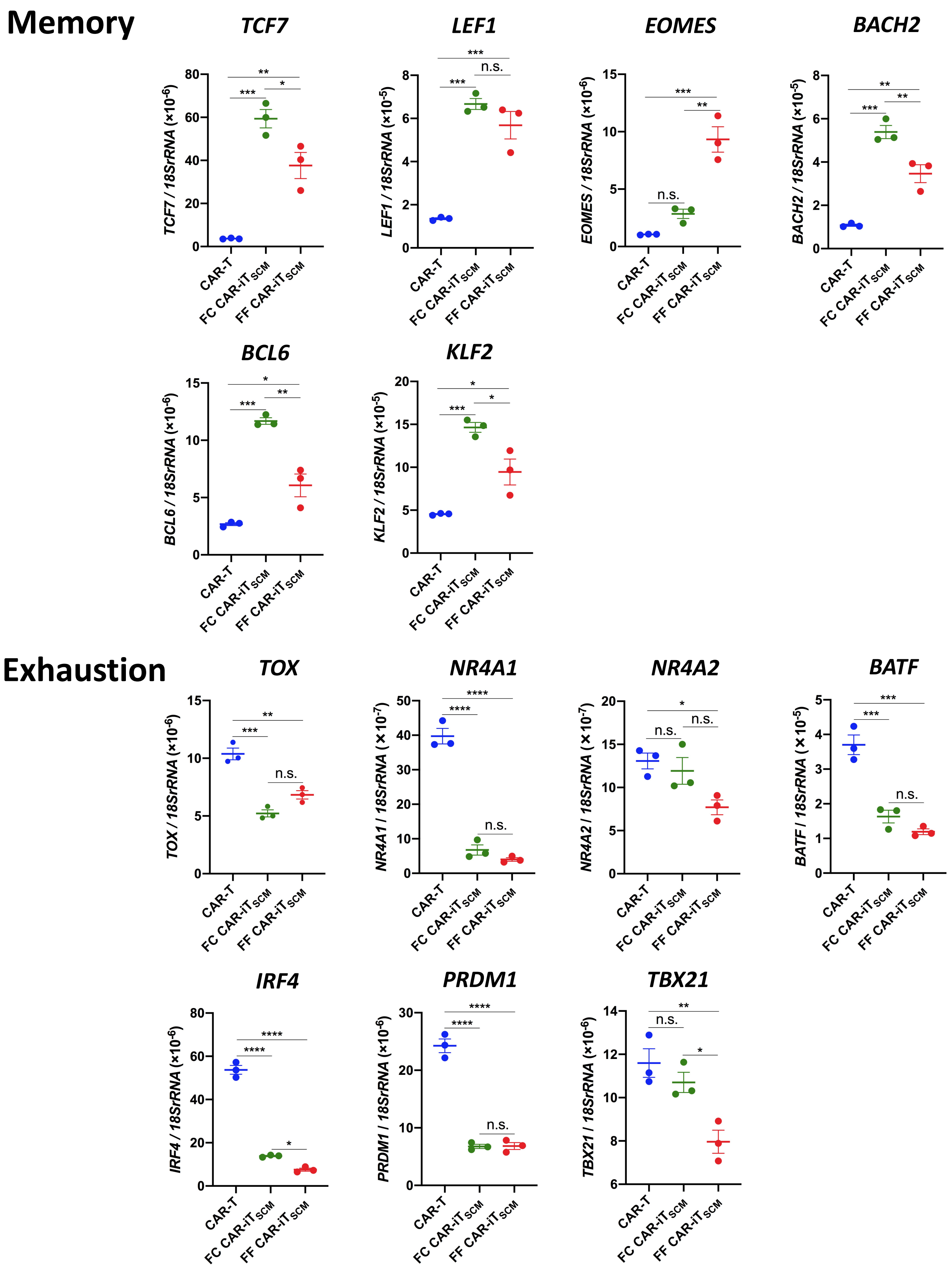

Supplementary Figure 4. Gene expression profiles of FF CAR-iT$_{SCM}$ cells as measured by quantitative PCR.

Gene expression of stem cell memory and memory-associated genes or exhaustion-associated genes in CAR-T cells, FC CAR-iT$_{SCM}$ cells, and FF CAR-iT$_{SCM}$ cells, as measured by quantitative PCR. Data are presented as mean ± SEM. *, $p < 0.05$; **, $p < 0.01$; ***, $p < 0.001$; ****, $p < 0.0001$; n.s., not significant; one-way ANOVA. Data are representative of at least two independent experiments.
