# Supplementary material for: Rejuvenating Effector/Exhausted CAR T Cells to Stem Cell Memory–Like CAR T Cells By Resting Them in the Presence of CXCL12 and the NOTCH Ligand
Source: Cancer Res Commun. 2021 Oct 19;1(1):41–55. doi: 10.1158/2767-9764.CRC-21-0034 (PMC9973402; doi:10.1158/2767-9764.CRC-21-0034)
Supplement: Supplementary Figure 5 — Stem cell memory gene profile and marker expression. [file crc-21-0034-s05.pdf]

Supplementary Figure 5

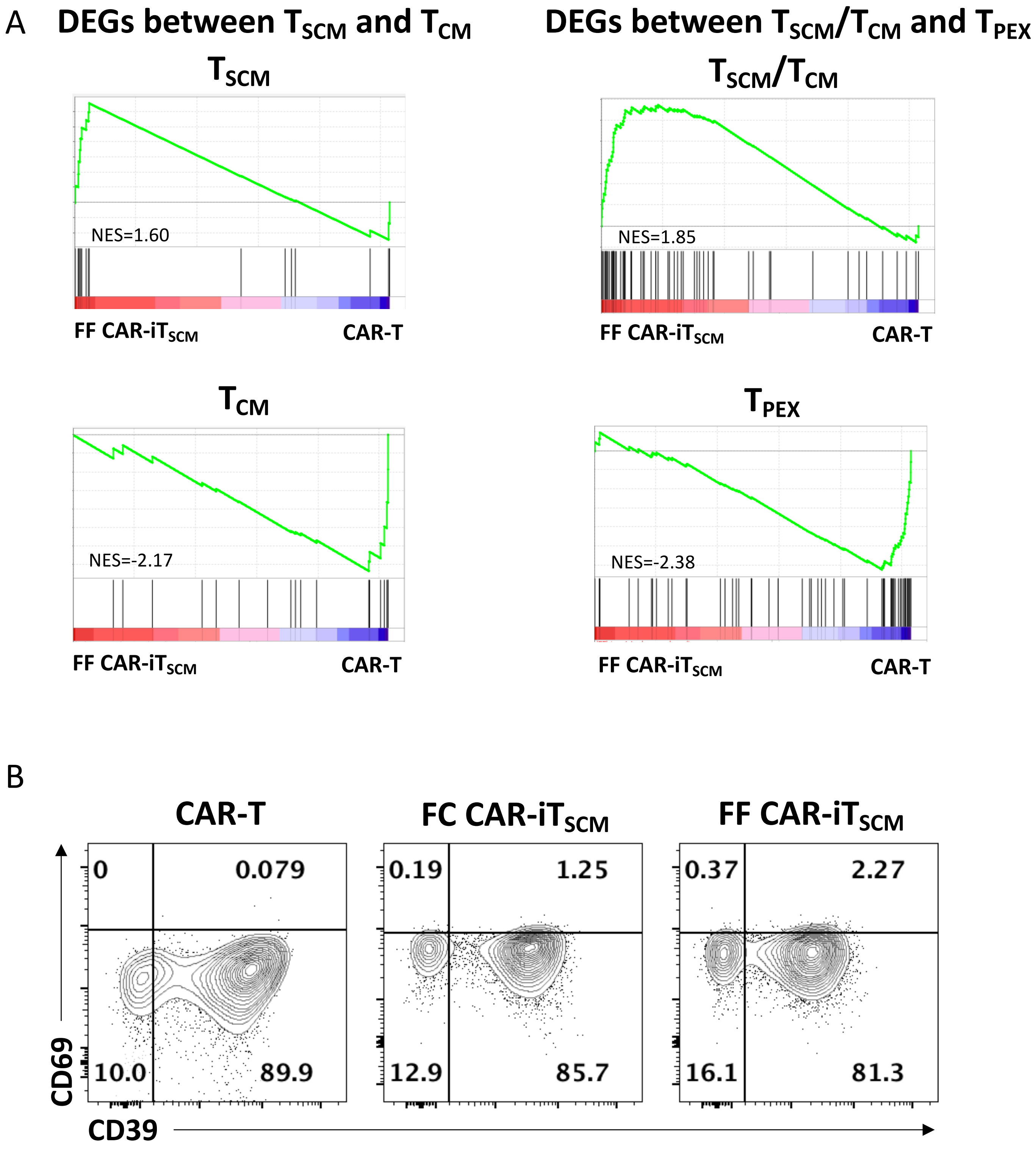

Supplementary Figure 5. Stem cell memory gene profile and marker expression.
(**A**) Gene set enrichment analysis performed using RNA-seq data for CAR-T and FF CAR-iT_{SCM} cells (gene set: DEGs between T_{SCM} and T_{CM} or T_{SCM}/T_{CM} and T_{PEX} as reported by Galletti et al., *Nat Immunol,* 2020[37]). (**B**) Representative FACS profile of CD39 and CD69 expression on CAR-T cells, FC CAR-iT_{SCM} cells, and FF CAR-iT_{SCM} cells.
