# Supplementary material for: Rejuvenating Effector/Exhausted CAR T Cells to Stem Cell Memory–Like CAR T Cells By Resting Them in the Presence of CXCL12 and the NOTCH Ligand
Source: Cancer Res Commun. 2021 Oct 19;1(1):41–55. doi: 10.1158/2767-9764.CRC-21-0034 (PMC9973402; doi:10.1158/2767-9764.CRC-21-0034)
Supplement: Supplementary Figure 6 — Metabolic state analysis of FF CAR-iTSCM cells. [file crc-21-0034-s06.pdf]

Supplementary Figure 6

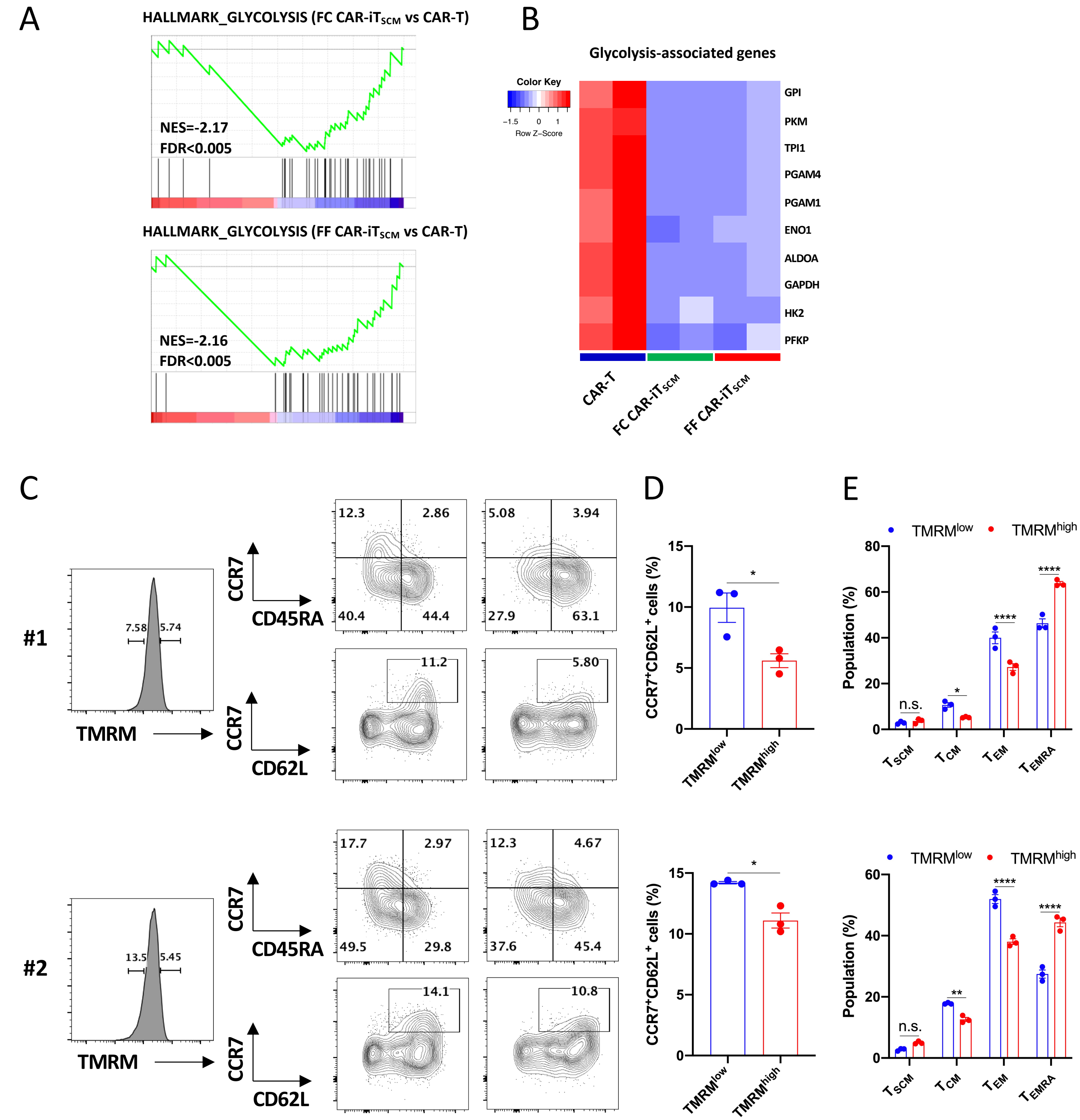

Supplementary Figure 6. Metabolic state analysis of FF CAR-iT$_{SCM}$ cells

(**A**) Gene set enrichment analysis of glycolysis (gene set: HALLMARK_GLYCOLYSIS) performed using PC1-driving genes between FC CAR-iT$_{SCM}$ cells and CAR-T cells (upper) or FF CAR-iT$_{SCM}$ cells and CAR-T cells (bottom). (**B**) Gene expression of glycolysis-associated genes in CAR-T cells, FC CAR-iT$_{SCM}$ cells, and FF CAR-iT$_{SCM}$ cells. (**C**) Representative FACS profile of the separation mitochondrial membrane potential (ΔΨm) for low or high cells from CD8^{+} CAR-T cells from two healthy donors (left). Representative FACS profile of CD45RA and CCR7 expression or CCR7 and CD62L expression in ΔΨm low or high CD8^{+} CAR-T cells from two healthy donors (right). (**D**) The percentage of CCR7^{+}CD62L^{+} cells in ΔΨm low or high CD8^{+} CAR-T cells from two healthy donors. (**E**) The population of T$_{SCM}$ (CD45RA^{+}CCR7^{+}), T$_{CM}$ (CD45RA^{-}CCR7^{+}), T$_{EM}$ (CD45RA^{-}CCR7^{-}), T$_{EMRA}$ (CD45RA^{+}CCR7^{-}) in ΔΨm low or high CD8^{+} CAR-T cells from two healthy donors. Data are presented as mean ± SEM. *, $p < 0.05$; **, $p < 0.01$; ****, $p < 0.0001$; n.s., not significant; Student's t-test (**D**) or one-way ANOVA (**F**). Data are representative of at least two independent experiments.
