# Supplementary material for: Rejuvenating Effector/Exhausted CAR T Cells to Stem Cell Memory–Like CAR T Cells By Resting Them in the Presence of CXCL12 and the NOTCH Ligand
Source: Cancer Res Commun. 2021 Oct 19;1(1):41–55. doi: 10.1158/2767-9764.CRC-21-0034 (PMC9973402; doi:10.1158/2767-9764.CRC-21-0034)
Supplement: Supplementary Figure 7 — Representative FACS profile of the mitochondrial membrane potential, cellular ROS, and DNA damage in FF CAR-iTSCM cells. [file crc-21-0034-s07.pdf]

Supplementary Figure 7

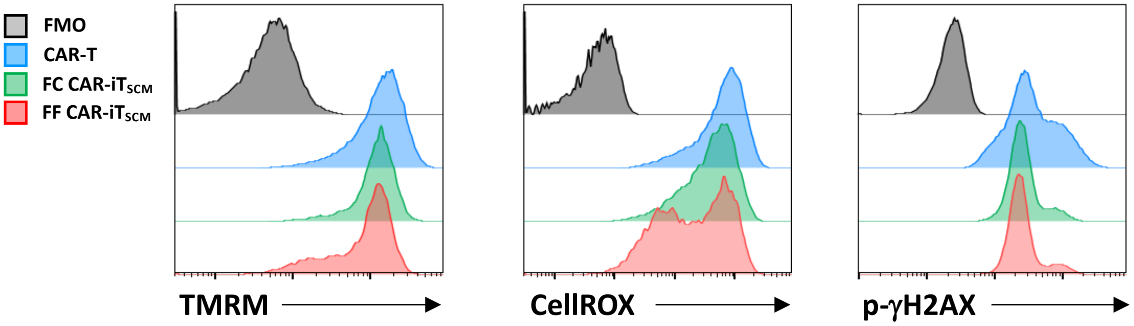

Supplementary Figure 7. Representative FACS profile of the mitochondrial membrane potential, cellular ROS, and DNA damage in FF CAR-iT$_{SCM}$ cells.

Representative FACS profile of the mitochondrial membrane potential using TMRM, cellular ROS using CellROX, and DNA damage using phospho-γH2AX in CAR-T, FC CAR-iT$_{SCM}$, and FF CAR-iT$_{SCM}$ cells. The gray histograms represent the fluorescent minus one as the controls.
